# An Integrated Study of *Toxocara* Infection in Honduran Children: Human Seroepidemiology and Environmental Contamination in a Coastal Community

**DOI:** 10.3390/tropicalmed5030135

**Published:** 2020-08-23

**Authors:** Sergio A. Hernández, José A. Gabrie, Carol Anahelka Rodríguez, Gabriela Matamoros, María Mercedes Rueda, Maritza Canales, Ronald Mergl, Ana Sanchez

**Affiliations:** 1 Department of Health Sciences, Brock University, St. Catharines, ON L3S 2A1, Canada; hernandez@brocku.ca (S.A.H.); jgabrie@brocku.ca (J.A.G.); gm18@brocku.ca (G.M.); 2 Department of Parasitology, School of Microbiology and Institute of Microbiology Research, National Autonomous University of Honduras, Tegucigalpa, Honduras; carol.rodriguez@unah.edu.hn (C.A.R.); maria.rueda@unah.edu.hn (M.M.R.); maritza.canales@unah.edu.hn (M.C.); 3 Niagara Falls Animal Medical Centre, Niagara Falls, ON L2E 6Z8, Canada; mergl.ron@gmail.com

**Keywords:** *Toxocara*, toxocariasis, zoonosis, seroepidemiology, neglected tropical diseases, Honduras

## Abstract

(1) Background: Infections caused by *Toxocara canis* and *T. cati* are considered zoonoses of global importance. Reports from North and South America indicate that human infections are widespread in both continents, but epidemiological information from Central America is still lacking. (2) Methodology: In the present cross-sectional multi-year study, we aimed to undertake the first seroepidemiological and environmental study on toxocariasis in Honduras. This included the determination of seroprevalence of anti-*Toxocara* spp. antibodies in children using a *Toxocara* spp. purified excretory-secretory antigens enzyme-linked immunosorbent assay (TES-ELISA) and a confirmatory Western blot. As well, through statistical analysis including logistic regression we aimed at identifying relevant biological and epidemiological factors associated with seropositivity. The study also entailed detection of parasites’ eggs in the soil samples both through Sheather’s concentration method and a nested polymerase chain reaction-restriction fragment length polymorphism (PCR-RFLP) method. (3) Results: The study was undertaken in a coastal community of Honduras in 2 different years, 2015 and 2017. A total of 88 healthy schoolchildren completed the study, with participation of 79% (73/92) and 65% (46/71) of the student body in 2015 and 2017, respectively. Thirty-one children participated in both years (i.e., dual participants). Through both serological tests, seropositivity was confirmed in 88.6% (78/88) of children. Due to the high number of seropositives, logistic regression analysis was not possible for most socio-economic and epidemiological variables. Eosinophilia, on the other hand, was associated with seropositivity, independently of other intestinal helminthic infections. Continued seropositivity was observed in most of the dual participants, while seroconversion was determined in 8 of these children. Microscopic examination of soil samples did not yield any positive results. Through nested PCR-RFLP, 3 of the 50 samples (6%) were positive for *Toxocara* spp.; two were identified as *T. canis* and one as *T. cati*. (4) Conclusions: This work documents for the first time, high levels of human exposure to *Toxocara* spp. in Honduras. These findings, along with the country’s favorable epidemiological conditions for this zoonosis, emphasize the need for more research to determine whether this infection is underreported in the country.

## 1. Introduction

*Toxocara* spp. are cosmopolitan zoonotic parasites that utilize dogs, cats, foxes and other canids and felids as definitive hosts. When harboring adult worms in their intestine, these animals extensively contaminate their surroundings with their stools containing parasite ova [1]. *Toxocara* species are distributed worldwide, with higher prevalence where infected domestic dogs and cats are allowed to defecate in public spaces [2]. Once fully developed in the environment, *Toxocara* eggs are infectious to definitive hosts as well as to humans. In the latter, however, the parasites do not reach adult stages but rather lodge in tissues as larval stages causing a wide spectrum of pathologies grouped under the clinical term toxocariasis (also called ‘toxocarosis’) [3].

The significance of human toxocariasis as a disease remains enigmatic, partly due to the multifaceted, nonspecific and cryptic nature of symptoms, making this an insidious disease more closely related to disability and infirmity than mortality. Further, toxocariasis can lead to significant and irreversible damage such as blindness and fibrotic lesions in visceral organs. Recent research suggests that this infection may partially account for cognitive deficits and other neurological complications seen among socioeconomically disadvantaged children [1,4]. There is a strong body of research from Europe and South America and a recent interest resurgence in the United States [5,6].

Conversely, the epidemiological situation of toxocariasis in Central America is largely unknown [7,8]. Even in Latin America and the Caribbean (LAC) nations, where other neglected tropical diseases (NTDs) are well-characterized, toxocariasis has not been consistently studied and no estimates of regional prevalence have been calculated [8]. Despite that data show that *Toxocara* is an important infection in dogs and probably in cats in Central America [9], a recent review by Ma et al. brings to light the paucity of research on human *Toxocara* infection in this particular geographic region [10].

Among Central American countries, Honduras is a country that, due to its climatic and socio-economic characteristics, is endemic for several NTDs and other infections [8]. With over 60% of the population living in poverty (i.e., earning < $2 USD/day) [11,12,13], and with a large uncontrolled population of domestic cats and dogs, the country offers optimal conditions for *Toxocara* spp. transmission; yet data on toxocariasis is almost non-existent [4,7,8].

In the present study, we aimed to undertake the first seroepidemiological and environmental study on toxocariasis in Honduras. Firstly, we set out to determine the seroprevalence of anti-*Toxocara* spp. antibodies in children as an indicator of exposure to the parasite. Secondly, we sought out to investigate potential associations between seroprevalence and relevant biological and epidemiological factors. Finally, we conducted an environmental sampling to confirm that soil in public spaces could be one source of infection for the study population.

## 2. Materials and Methods

### 2.1. Study Design and Population

The present investigation was designed as an exploratory, cross-sectional study. A non-probability, purposive sampling method (based on expert knowledge of the population) was used to obtain the study sample. A minimum sample size was not calculated. Rather, research participants were recruited from a primary school population with high prevalence of soil-transmitted helminth (STH) infections. Two data collection visits took place: in August 2015 and October 2017.

The study was conducted in the village of Santa Cruz del Junco, within the municipality of Tela, department of Atlántida, on the northern Caribbean coast of Honduras.

The municipality has an area of 1196 km^2^ and an elevation of 3 m above sea level. The most recent census (2013) by the Honduran National Institute of Statistics recorded a population of 96,758 inhabitants, of which 48.8% were rural residents and 32.8% were ≤14 years old. The same census also documented a poverty index of 51%, with 42% of Tela’s inhabitants listing agriculture, animal husbandry or fishing as their main source of income [14]. According to the Köppen Climate Classification, Tela exhibits a tropical rainforest climate, characterized by a lack of dry or wet seasons, as all months present at least 60 mm of precipitation; a condition that contributes to high levels of humidity. There is also no defined summer or winter in Tela, and it is typically hot and wet year-round [15].

The city of Tela, named after the municipality, is located between 15°47′00″ North latitude and 87°28 00″ West longitude, placing it approximately 67 km north-east of the city of San Pedro Sula, the primary industrial center in Honduras and the nation’s second largest city after the capital [14]. The study village is located approximately 11 km from the city’s center.

A national survey investigating prevalence and intensities of soil-transmitted helminths (STH) in Honduran schoolchildren had identified Tela as an area with an STH prevalence close to 50% [12]. These conditions are ideal for the transmission of a variety of parasites, including *Toxocara* spp. [16,17].

### 2.2. Study Sample

Schoolchildren from the village’s only public primary school (consisting of grades one through six), were invited to participate in the study, with no exclusion criteria. We focused on school-age children to determine exposure to *Toxocara* spp. based on their known contact with soil and propensity to harbor STH more than adults [8]. It is widely known that *Toxocara* infection is markedly more associated with children, a finding related to certain determinants such as geophagia, playing habits and hygienic practices [18,19,20].

### 2.3. Data Collection

Each participant’s parent/guardian partook in a face-to-face interview that used a pre-developed questionnaire as a guideline for data collection. The questionnaire was structured into different categories aiming to gather information including demographic and epidemiological data, domestic animal presence in the household, relevant patterns of child behavior and knowledge of parasites.

### 2.4. Blood Sample Collection and Analysis

Sera collected from each participating child was tested for the detection of anti-*Toxocara* IgG antibodies by way of a commercially available ELISA kit (Diagnostic Automation, Inc./Cortez Diagnostics, Calabasas, CA, USA). According to manufacturer instructions, any sample that yielded an absorbance ≥ 0.3 optical density (OD) units was considered seropositive. All positive ELISA results were confirmed by a Western blot (WB) assay, as well as 3 out of the 8 ELISA-negative samples. As per manufacturer instructions, the presence of two or more bands of low molecular weight were regarded as a positive result (LDBIO Diagnostics, Lyon, France). All assays were conducted according to manufacturer instructions. During the 2015 phase of the study, an eosinophil count was performed by an independent laboratory (Hospital CEMESA clinical laboratory), located in San Pedro Sula city, 1 h-drive from the study site. Eosinophilia was defined as ≥500 eosinophils/µL [21].

### 2.5. Stool Sample Collection and Analysis

In order to rule out potential serum cross-reactivity due to *Ascaris lumbricoides* infection as well as other parasitic infections characterized by eosinophilia, we analyzed children’s stool samples for STH. A single stool sample was collected from each child and analyzed the same day at the local hospital laboratory using the Kato-Katz technique (Vestergaard Frandsen, Lausanne, Switzerland). With a Kato-Katz template delivering 41.7 mg of sample, we used a factor of 24 to obtain the number of eggs per gram (epg) of stool. Kato-Katz smears were microscopically examined after 30 min of clarifying time. Any insufficient or unsuitable (not formed) stool sample was analyzed by direct wet mount examination.

### 2.6. Soil Sample Collection and Analysis

A total of 50 soil samples, each approximately weighing 30 g, were collected from 5 different sites in the community of Santa Cruz del Junco in 2017 (10 samples per site). Samples were collected from the superficial layer of soil, up to a depth of 5 cm, avoiding any pebbles or grass. Soil samples were exported according to international regulations to Brock University in Canada for further microscopic and molecular analysis (permit number P-2018-00845).

Each sample was dried overnight at 37 °C, sieved with a 150 µm pore size sifter and the resulting refined soil was kept at room temperature in 50 mL conical tubes. Volumes obtained through this process varied from 3 to 13 mL.

The detection of *Toxocara* spp. in the refined samples, was done through a centrifugation/passive flotation technique (i.e., Sheather’s) and a nested PCR-RFLP. For the Sheather’s technique, samples were analyzed in duplicates as follows: 1 g of soil was transferred to a 15 mL centrifuge tube, mixed with 9 mL of distilled water, vortexed for one minute and then centrifuged at 1500× *g* for 5 min. The resulting supernatant was discarded, and a flotation sucrose solution (Specific Gravity, SG∼1.27) was added to the pellet to complete a volume of 10 mL. Tubes were vortexed and filled with the same sucrose solution until a positive meniscus formed. Cover slips were placed on top of the tubes and, after a 60-min period of passive flotation, were examined microscopically at 10× and 40× magnifications for the presence of *Toxocara* spp. eggs.

For the molecular detection of *Toxocara* spp., DNA was extracted from a 250 mg aliquot of the refined soil using a commercial kit (Norgen Soil DNA Isolation Plus Kit cat# 64040, Norgen Biotek Corp., Thorold, ON, Canada). Extraction was performed according to the manufacturer’s protocol, with two modifications: (i) a preparatory step consisting of thermal stress, and (ii) a substitution of the beads provided with the kit. The thermal stress procedure entailed three rounds of a freeze-heat cycle, whereby samples were placed at −80 °C for 10 min followed by dry incubation at 90 °C for 10 additional minutes. This thermal stress step was followed by overnight incubation with proteinase K at 56 °C. For the second modification, the plastic beads in each bead tube were replaced with a medley of sterile stainless-steel beads measuring 3 mm, 2 mm and 1.5 mm.

A nested PCR approach, targeting segments of the 18S rRNA gene, was performed using the following primers: PCR 1: NC5f 5′–GTAGGTGAACCTGCGGAAGGATCATT–3′ and NC2r 5′–TTAGTTTCTTTTCCTCCGCT–3′ and PCR 2: FM1f 5′-TTGAGGGGAAATGGGTGAC-3′ and FM2r 5′–TGCTGGAGGCCATATCGT–3′. Each PCR mix contained 10 µM for both forward and reverse primers and 2 µL of template DNA, with a final volume of 25 µL. The cycling conditions for PCR 1 were as follows: 94 °C for 3 min; followed by 35 cycles of 94 °C for 45 s, 60 °C for 1 min, 72 °C for 1 min and a final extension of 72 °C for 6 min. For PCR 2, the cycle involved 94 °C for 3 min; 35 cycles of 94 °C for 30 s, 60 °C for 30 s and 72 °C for 1 min and a final extension of 72 °C for 5 min. The PCR was optimized for the detection of DNA originating from a single egg using spiked soil samples as well as soil negative controls.

Two restriction enzymes, SalI and Mval, were used to differentiate between *T. canis* and T. *cati* [13]. For this, amplicons were digested at 37 °C for 3 h. Digestion of the product by SalI 1500 U (ThermoScientific ER0641, ThermoFisher Scientific, Waltham, MA, USA) produced 2 fragments of ~320 bp and ~394 bp in *T. canis* and an undigested band of 736 bp for *T. cati*. While digestion by Mval (BstNI) 2000 U (ThermoScientific ER0551, ThermoFisher Scientific) yielded 2 fragments of ~600 bp and ~100 bp in *T. cati* and an undigested band of ~700 bp for *T. canis*. Both PCR products and digested fragments were separated in 1.5% agarose gels with ethidium bromide.

### 2.7. Statistical Analyses

Data were inputted into a Microsoft Excel 2016 spreadsheet (Microsoft Corp., Redmond, WA, USA), cleaned for errors or missing values and exported for analysis to the STATA 13 software package (StataCorp LP., College Station, TX, USA).

For characterization of the study population, we used descriptive statistics for both continuous and categorical variables. Seroprevalence of anti-*Toxocara* antibodies was calculated with the confirmatory test. Assessment of the significance of univariate associations with positive serology (as confirmed by WB) was done by Fisher’s exact test. A logistic regression model was applied to investigate the relationship between the various epidemiological risk factors with anti-*Toxocara* antibody presence. Odds ratios (OR) were determined with 95% confidence intervals (CI).

Sensitivity, specificity, positive predictive, and negative predictive values were calculated for the ELISA, compared to the Western blot. The kappa statistic for level of agreement between both diagnostic tools was also evaluated. The level of agreement was interpreted as moderate, strong or almost perfect if the value of kappa was 0.60–0.79, 0.80–0.90 or >0.90, respectively [22]. For the eosinophilia measured in the first phase of the study, a Mann–Whitney *U* test was employed to determine if there was a significant difference in circulating eosinophil counts in seropositive vs. seronegative individuals. Level of significance was established at α = 0.05.

### 2.8. Ethics Statement

Both phases of the present study received ethics clearance from both participating institutions, in Canada (Brock University; file number: 17-032-Sanchez, clearance received 12 September 2017; file number: 14-224-Sanchez, clearance received: 8 May 2015) and Honduras (National Autonomous University of Honduras, Tegucigalpa M.D.C, Honduras. Committee of Ethics, Master’s program in Zoonotic and Infectious Diseases School of Microbiology; file number: 04-2017, clearance received: 21 September 2017; file number: 01-2015, clearance received: 1 August 2015). In addition, approval for the implementation of this study was requested from the participating school’s principal and grade teachers. Both parental consent and children’s assent were also required prior to an individual’s participation.

## 3. Results

### 3.1. Study Participation and Characterization of the Study Population

A total of 88 different children completed the study. Enrolments were 73 and 46 for 2015 and 2017, respectively, but there were 31 children coincidentally enrolled in both years. These participants were deemed “dual participants”. Study participation varied between years: in 2015, participation was 79% of the school enrolment (73/92) whereas for 2017 it was only 65% (46/71). The reason for the latter was a widespread outbreak of hemorrhagic conjunctivitis in Honduras (and throughout Latin America and the Caribbean) due to coxsackievirus A24, which prevented children’s attendance to school and their potential enrolment in the study.

The final study sample was comprised of students between the ages of 6–15 years (mean 9.83 years ± 2.25) and 45 (51.1%) girls. A summary of the epidemiological and serological data and children’s behavior is displayed in Table 1. An important proportion (71.6%) of interviewed participants reported dog ownership by their family (Table 1). Additionally, most participants with household pets, either dogs or cats, specified that these animals strayed from the household freely (72.6% and 66.7%, respectively). Among the behaviors listed, it is important to mention that 80.4% of children said they had some sort of contact with soil, mostly with soil from the playground adjacent to the school.

### 3.2. Seroprevalence of Anti-Toxocara Antibodies

Of the 88 serum samples, 80 were ELISA-positive. Western blot results (WB) confirmed that 78 out of the 80 sera were indeed positive for anti-*Toxocara* antibodies and hence, an overall seroprevalence of 88.6% was documented (Table 1). As a manner of internal quality control, we tested with WB 3 out of the 8 (37.5%) ELISA-negative samples. The WB confirmed the absence of specific antibodies in these 3 samples. This was in addition to including the kit’s negative controls.

Of the seropositive participants, 52.6% (41/78) were males, but neither the univariate analysis nor the logistic regression model identified male sex as statistically significant for seropositivity (*p* = 0.09; OR = 4.43, 95% CI = 0.87–22.42, *p* = 0.07). Due to the high number of children with positive serology (the primary outcome), logistic regression analysis was not possible for most variables. Table 2 shows the results of this analysis for the remaining variables none of which were identified as statistically significant. The results of the dual participants are demonstrated in Table 3. It can be seen that the proportion of seropositive children increased, as 8 of the 31 dual participants seroconverted in the two-year interim (Table 3).

### 3.3. Toxocara spp. Seropositivity and Eosinophilia

The eosinophil count performed in 2015 revealed eosinophilia (defined as ≥ 500 eosinophils/µL) in 16 children out of the 73 participants (21.9%). A Mann–Whitney U test was done to see if there was a significant difference in the count of circulating eosinophils in schoolchildren who tested seropositive compared to those seronegative (Table 4). To account for possible confounders, the test was also applied when controlling for individuals with any kind of STH infection and *T. trichiura* specifically. A significant difference was found between both subgroups (Table 4). For those that were found free of any STH infection, *Toxocara*-seropositive children averaged 191.3 eosinophils/µL, compared to 101.2 eosinophils/µL in those who were seronegative (*p* = 0.058). Increased eosinophil levels were also documented for those without trichuriasis (*p* = 0.035) (Table 4).

### 3.4. Comparison of Serodiagnostic Techniques

As an additional step, the performance of the TES-ELISA test used in this study was measured against the Western blot, the current recommended confirmatory test for anti-*Toxocara* antibody detection. The average ELISA sensitivity was 100% (95% CI 95.4–100%) and the average specificity was 80% (95% CI 44.4–97.5%), lower than the 93.7% reported by the manufacturer. With these two parameters, a kappa (κ) statistic was calculated to establish the degree of agreement between the two serodiagnostic tests. In this case, the resulting kappa statistic was κ = 0.87, an indicator of a strong agreement between the two diagnostic tools. Additionally, the average ELISA’s positive and negative predictive value (PPV) were 97.5% (95% CI: 91.3–99.7%) and 100% (95% CI: 63.1–100%), respectively.

### 3.5. Intestinal Parasitic Infections

Two children provided insufficient or unsatisfactory stool samples for the Kato-Katz technique, so the samples were instead analyzed by direct wet mount. Overall, 34 of 88 (38.6%) children were infected with at least one STH species of which *T. trichiura* was the most prevalent (30/88 or 34.1%). Concurrent parasitoses were observed in 11 (12.5%) participants, highlighting the possibility of potential cross-reacting antibodies (Table 1).

### 3.6. Identification of Toxocara spp. in Soil Samples

Microscopic examination after sucrose concentration of soil samples did not yield any positive results. However, eggs of *T. trichiura* and *A. lumbricoides* were detected with this method in 3 (6%) samples.

In contrast, in the nested PCR-RFLP we identified 3 of the 50 samples (6%) as positive for *Toxocara* spp.; two were identified as *T. canis* and one as *T. cati* DNA. These 3 samples were collected in two of the five collection sites, one of which turned out to be the playground adjacent to the school. These *Toxocara* positive samples were negative for any STH by microscopy after the Sheather’s concentration technique.

## 4. Discussion

Adding to the list of neglected tropical diseases (NTDs) in Honduras, this work documents for the first time, high levels of human exposure to *Toxocara* spp. in the country, and suggests the potential for this infection to be seriously underreported.

To our knowledge, only one clinical case of toxocariasis has been published in Honduras. Puerto-Sanabria et al. [23] described in 2016 a case in a 14-month-old infant with central nervous system involvement. Canine toxocariasis is poorly documented as well, even though the infection is frequently treated at veterinary clinics (Sanchez A, personal observations). Reference to the infection circulating in Honduran puppies is made in a 2002 review paper by Javier and Alger [24]. In addition, through an exhaustive search of Latin American databases and Honduran journals, we were able to find two publications: a study published in the Honduran Medical Journal reporting a toxocariasis prevalence of 3.8% in a sample of 207 dogs (82 owned, 69 from a kennel, and 56 free-roaming) [25], and a conference poster presentation by Valle-Ramirez & collaborators describing *T. canis* infection in 12% of 177 dogs examined [26].

Other than the publications mentioned above, we were not able to find more toxocariasis-related data either published or in the grey literature. Nonetheless, given that canine and human toxocariasis are prevalent in countries with similar climatic and socio-economic characteristics [6], we theorized that this parasitic disease is highly prevalent in Honduras.

The study findings confirmed our hypothesis —at least in the study sample—as we determined an overall seroprevalence of 88.6%. This is a surprisingly high seropositivity, but with the use of a confirmatory test (i.e., Western blot), we are confident that our results are reliable. Such high seroprevalence warrants a clinical investigation as covert toxocariasis would be a serious concern among the studied children [3]. Further, evidence indicates strong links between seropositivity and cognitive and developmental delays [1,4,5,27,28]. To reinforce the plausible link between seropositivity and health effects, we found that after controlling for STH infections, the presence of anti-*Toxocara* antibodies was associated with high levels of eosinophilia. In fact, of 73 children tested in 2015, 16 had high counts of circulating eosinophils and all were seropositive. A difference was observed between the eosinophil geometric means of seropositive children when compared to those of seronegative ones (191.3 eosinophils/µL vs. 101.2 eosinophils/µL, respectively), but this difference did not reach statistical significance (*p* = 0.058).

Many studies have already pointed out eosinophilia as a clinical marker for several helminthic and some protozoal infections [21]. Among helminthic infections, strongyloidiasis is commonly associated with strong eosinophilic reactions, whereas ascariasis and trichuriasis are known to be eosinophilia-inducing, albeit to a lesser degree [29,30,31]. *A. lumbricoides, T. trichiura*, and hookworm infections showed a pattern of endemic transmission among the studied children, with an overall prevalence of 38.6% across the 2-year period. The higher prevalence of trichuriasis is noteworthy—a fact that we have demonstrated in other Honduran communities and that had prompted us to investigate anthelminthic resistance in light of decades of deworming campaigns in the country [32].

In Honduras, strongyloidiasis is not reported frequently, especially in children [12]. Other parasitoses characterized by eosinophilia such as trichinellosis, filariasis, fascioliasis, echinococcosis, schistosomiasis, etc. [21,29,31,32,33,34,35,36] are not prevalent in the country.

Eosinophilia, on the other hand, has long been recognized as an important biomarker for toxocariasis [37,38,39,40]. In the major clinical presentation, visceral larva migrans (VLM) syndrome, eosinophilia is a distinguishable biomarker that can be drastically elevated [37,41]. In other clinical presentations for instance, ocular larva migrans (OLM), covert toxocariasis or neurotoxocariasis, eosinophilia can still be present, but on average, at lower levels than in VLM [20,37,42]. The concurrent findings of eosinophilia and seropositivity among the studied children are consistent with results from other studies [43,44,45,46]. Moreover, the combination of eosinophilia and seropositivity among the studied children may suggest the presence of an active infection (i.e., with viable larvae in tissues) [47]. A clinical and laboratory examination of these children could elucidate whether their seropositive status can be linked to covert (inapparent) or common toxocariasis [3].

Gender of the participating children was not identified as a statistically significant risk factor in our study either. It is interesting to note, however, that the proportion of seropositive boys (41/45, 95.35%) was higher than that of girls (37/45, 82.22%), and that the logistic regression analysis identified boys at about 4-fold increased odds of having anti-*Toxocara* antibodies. It is generally speculated that this association is related to boys’ increased exposure to infective eggs in the soil from outdoor activities as well as their proclivity to play and have more contact with animals than girls [48,49].

In terms of epidemiological factors associated to seropositivity, we aimed to identify those postulated in the literature (e.g., soil contact, geophagia, dog ownership, raw beef consumption) but unfortunately, the substantial levels of seropositivity within such a small sample prevented us from detecting statistically significant associations, notably between plausible variables such as soil contact, dog ownership, undercooked beef consumption, or geophagia.

In fact, with almost all children (95%) reporting frequent exposure to soil, and almost 90% of them being seropositive, it would have been surprising to detect any statistical association between these two variables. Still, despite the lack of statistical significance, our logistic regression analysis detected that those reporting soil contact were twice as likely to have anti-*Toxocara* spp. antibodies.

We were also unable to identify the consumption of raw fruits or vegetables as a significant risk factor for anti-*Toxocara* spp. antibody presence. Yet, children who affirmed these dietary habits were found to be at four-times greater odds of being seropositive. A great proportion of children who admitted consuming raw fruits/vegetables were found to be seropositive (34/35, 97.14%).

Lastly, in terms of risk factors, even though the association was not significant, most children reporting family dog ownership (57/63) had almost twice the odds of being seropositive compared to their counterparts. This finding may reflect the considerable impact of these canine populations as a source of contamination, especially for children [50,51,52]. This is particularly true in areas such as the one studied here, where we observed free-ranging dogs running rampant with unrestricted access to public spaces. It would be important to conduct a canine seroepidemiological survey in the community—and in the country at large—to ascertain the size of the dog population and get an overview of the prevailing health issues including zoonotic pathogens such as toxocariasis.

A unique aspect of the present investigation is the testing of the study population in two different years, which allowed for data collection within the same community at two separate time points (2015 and 2017). Moreover, since the second phase of this study (2017) was carried out within the very same school as two years prior, overlap of some participants was inevitable. Initially, 21 of 31 (67.7%) dual participants tested seropositive via TES-ELISA in 2015. In 2017, this proportion increased to 30 of the 31 (96.7%). Of these 9 seroconverted participants, the confirmatory Western blot identified one as a false positive, leading to the conclusion that 8 children had been exposed to the parasite during the two-year interim. Seroconversion may be attributed to changes in social and recreational behaviors of children as they age. In the two-year study gap, these children could have experienced dietary changes or new/more frequent contact with definitive or paratenic hosts, contaminated environments or fomites, or acquisition or strengthening of unhygienic habits (e.g., geophagia, inadequate handwashing, etc.) [2,18,53]. Some authors suggest that seroconversion is not intrinsically related to behavior changes. They propose that an increase in antibody titers to detectable levels might be due to the cumulative effect of persistent exposure and infection, or to the constant antigenic stimulation elicited by live larvae in tissues [1]. While in the studied population all these scenarios are possible, the fact remains that there is in the community a continuous presence of infectious sources.

Finally, to further investigate potential sources of infection, we integrated into the study an environmental component. Out of the 50 soil samples collected from 5 community sites, 3 samples (6%) from two sites contained *Toxocara* spp. eggs. Two of the three positive samples were collected from the playground adjacent to the school; an unfenced area that is heavily trafficked by children and animals, even outside school hours. In agreement with worldwide literature [54], our data suggest that the playground could be an important source of exposure to *Toxocara* spp. and if so, installing a fence around its perimeter would help mitigate the risk of exposure.

There are some limitations and strengths to this study. An important limitation that prevents generalization is the small sample size restricted to one cohort of schoolchildren. Not only did the study lack statistical power, but a cluster effect most certainly led to capturing a high prevalence phenomenon. Naturally, a small sample size and the extremely high seropositivity observed hindered meaningful statistical analyses. A second caveat worth mentioning pertains to the lack of clinical data from the studied children. Without investigating the potential health implications of a seropositive status, our study can only suggest that the high levels of exposure to *Toxocara* in this community underscore the need for appropriate attention from the health and veterinary sectors. One more limitation entails not including a canine survey. Although this was considered, we could not secure trained personnel to capture free-ranging dogs to obtain blood and stool samples.

Limitations notwithstanding, the study draws strength from the use of both an ELISA and a confirmatory Western blot, which lends reliability to our seroprevalence data. In addition, we were successful in detecting through PCR *Toxocara* spp. eggs in soil samples of 2 out of 5 collection sites. Further, we were able to identify both *T. canis* and *T. cati* in soil samples, opening research possibilities for the study of feline populations as well. A unique strength of the study is having a sub-sample of participants enrolled in both 2015 and 2017. This allowed us to run a nested comparison at two different time points and identify continued seropositivity as well as seroconversion.

In conclusion, we here present the first serological survey on human toxocariasis in Honduras, filling an important knowledge gap not only in the country but in the Central American region. Next steps should include conducting larger epidemiological, veterinary and clinical investigations using a One Health approach. Such data would inform an initial assessment of the burden of this neglected—almost invisible—zoonotic disease.

## Figures and Tables

**Table 1:** Study sample characteristics and laboratory results by year of study. Since there were 31 children who participated in both years, the final sample size amounts to a total of *n* = 88.

Characteristics	2015 *n* = 73 (%)	2017 *n* = 46 (%)	Total *n* = 88 (%) ^θ^
Males	35 (48%)	24 (52.2%)	43 (48.9%)
Females	38 (52%)	22 (47.8%)	45 (51.1%)
Age (years), *mean (SD)*	10.3 (1.98)	9.40 (2.25)	9.83 (2.25)
**Soil Transmitted Helminthiases (STH) Profile**			
Overall STH prevalence	30 (41.1%)	10 (21.7%) ^‡‡^	34 (38.6%) ^‡‡^
*Ascaris lumbricoides* infection	10 (13.7%)	2 (4.3%) ^‡‡^	10 (11.4%) ^‡‡^
*Trichuris trichiura* infection	26 (35.6%)	10 (21.7%) ^‡‡^	30 (34.1%) ^‡‡^
Hookworm infection	6 (8.2%)	0 (0%) ^‡‡^	6 (6.8%) ^‡‡^
Polyparasitic infections	13 (17.8%)	2 (4.3%)	11 (12.5%)
Awareness of STH	57 (78.1%)	24 (52.2%)	62 (70.5%)
Recalled having STH infection	48 (65.7%)	24 (52.2%) ^§^	54 (71.1%) ^§^
**Anti-*Toxocara* Antibody Serology and Eosinophilia**			
Positive by TES-ELISA	56 (76.7%)	45 (97.8%)	80 (90.9%)
Confirmed by Western Blot	56 (100%)	43 (95.6%)	78 (97.5%)
Eosinophilia (≥500 eosinophils/µL) *	16 (21.9%)	NA	16 (21.9%) *
**Domestic Animal Conditions**			
Dog ownership (*n* = 88)	53 (72.6%)	34 (73.9%)	63 (71.6%)
Free-ranging owned dogs	39 (73.5%)	23 (67.6%) ^+^	45 (72.6%) ^+^
Cat ownership (*n* = 46)	NA	24 (52.1%) ^†^	24 (52.1%) ^†^
Free-ranging owned cats	NA	16 (66.7%) ^†^	16 (66.7%) ^†^
**Children’s Behavior** ^†^			
Contact with soil in the village	NA	37 (80.4%)	37 (80.4%)
Contact with soil in school playground	NA	41 (91.1%) ^««^	41 (91.1%) ^««^
Geophagia	NA	2 (4.3%)	2 (4.3%)
Onychophagia	NA	11 (23.9%)	11 (23.9%)
Thumb-sucking	NA	4 (8.7%)	4 (8.7%)
Consume undercooked beef	NA	21 (45.6%)	21 (45.6%)
Consume raw fruits/vegetables	NA	35 (79.5%) ^◊◊^	35 (79.5%) ^◊◊^

NA: data not collected; * data for 2015 only; ^†^ data collected for 2017 participants only; ^‡‡^ two children did not provide satisfactory stool samples for Kato-Katz examination; ^§^ data not recalled for 10 children; ^+^ data not recalled for one child; ^◊◊^ data not recalled for two children; ^««^ data not recalled for one child; ^θ^ 31 dual participants counted only once. TES-ELISA: *Toxocara* spp. purified excretory-secretory antigens enzyme-linked immunosorbent assay

**Table 2:** Logistic regression analysis of *Toxocara* spp. seropositivity among the studied schoolchildren (*n* = 88).

Variable	Odds Ratio (OR)	95% Confidence Interval (CI)	*p* Value
Gender (Male)	4.43	0.87–22.42	0.072
Soil Contact	2.19	0.17–27.96	0.547
Onychophagia	0.61	0.04–7.61	0.698
Raw Beef Consumption	--	--	--
Raw Fruit/Vegetable Consumption	4.25	0.23–78.01	0.330
Dog Ownership	1.80	0.46–7.10	0.396
Dog Age ≤ 1 Year	--	--	--
Playground Contact with Soil	--	--	--

(--): Variable omitted from final model.

**Table 3:** Serological status of “dual participants”: schoolchildren who participated in both 2015 and 2017.

Participants	ELISA Positives 2015 *n* (%)	Western Blot Confirmed 2015 *n* (%)	ELISA Positives 2017 *n* (%)	Western Blot Confirmed 2017 *n* (%)	Seroconverted by 2017 *n* (%)
Males (*n* = 15)	12 (80%)	12 (80%)	15 (100%)	15 (100%)	3 (20%)
Females (*n* = 16)	9 (56.3%)	9 (56.3%)	15 (93.7%)	14 (87.5%)	5 (31.3%)
Total (*n* = 31)	21 (67.7%)	21 (67.7%)	30 (96.7%)	29 (93.5%)	8 (26%)

**Table 4:** Mann–Whitney U test results comparing geometric mean of circulating eosinophil levels in seropositive vs. seronegative schoolchildren (*n* = 73) ^†^.

Geometric Mean (G-Mean) Value	Western Blot Seropositives (95% CI)	Western Blot Seronegatives (95% CI)	*p* Value
G-Mean * eosinophils/µL	262.1 (211.7–324.4)	101.2 (59.6–171.9)	0.004
G-Mean eosinophils/µL (without STH)	191.3 (141.7–258.2)	101.2 (59.6–171.9)	0.058
G-Mean eosinophils/µL (without *T. trichiura*)	198.5 (151.2–260.5)	101.2 (59.6–171.9)	0.035

^†^ Eosinophilia data only collected in 2015; * G-Mean: geometric mean.
